# Photo-CIDNP in Solid State

**DOI:** 10.1007/s00723-021-01322-5

**Published:** 2021-04-06

**Authors:** Jörg Matysik, Yonghong Ding, Yunmi Kim, Patrick Kurle, Alexandra Yurkovskaya, Konstantin Ivanov, A. Alia

**Affiliations:** 1 Institut für Analytische Chemie, Universität Leipzig, Linnéstr. 3, 04103 Leipzig, Germany; 2 International Tomography Center, Institutskaya, 630090 Novosibirsk, Russia; 3 Institut für Medizinische Physik und Biophysik, Universität Leipzig, Härtelstr. 16-18, 04107 Leipzig, Germany; 4 Leiden Institute of Chemistry, Leiden University, Einsteinweg 55, 2333 CC Leiden, The Netherlands

## Abstract

Photo-CIDNP (photo-chemically induced dynamic nuclear polarization) refers to nuclear polarization created by the spin-chemical evolution of spin-correlated radical pairs (SCRPs). This phenomenon occurs in gases, liquids and solids. Based on the solid-state photo-CIDNP effect observed under magic-angle spinning (MAS), photo-CIDNP MAS NMR has been developed as analytical method. Here we report the origin, the theory and the state of the art of this method.

## Introduction

Chemically Induced Dynamic Nuclear Polarization (CIDNP) is a phenomenon in spin-chemistry [1–3], which is a sub-field of chemistry dealing with the role of spin degrees of freedom in chemical reactions. Spin-chemistry phenomena originate from the fact that many chemical reactions are electron-spin selective, i.e., they occur at different rates for reactants in different electron spin states. The most common example of such spin selectivity is given by radical pairs, reacting at different rates from the singlet and triplet states. In such cases, the reactivity is modulated by inter-conversion between these states. In turn, inter-conversion can be sensitive even to subtle effects of the nuclear spins. The effect of nuclear spins on chemical reactions is typically not expected, since the energy associated with spin state changes is extremely small, however, the unexpected discoveries of CIDNP and the magnetic isotope effect clearly demonstrate their crucial role. One more feature of CIDNP is that non-thermal populations of the reaction products generated in the course of radical pair recombination give rise to strong NMR signal enhancements. Hence, CIDNP is not only a spin-chemistry related phenomenon but it also belongs to the family of spin hyperpolarization methods. A similar phenomenon is optical nuclear polarization (ONP) but here a physical process involving excited triplet states occurs rather than a chemical reaction involving radical pairs (see below).

Observation of CIDNP by liquid-state NMR was indeed an absolutely unexpected discovery: it took a considerable amount of time to demonstrate conclusively that the effect was not an artifact and to explain it properly. Accounts of the early CIDNP experiments have been reported by Joachim Bargon [4] and Robert Kaptein [5]. In this review, we want to report the history of the solid-state photo-CIDNP effect. CIDNP effects in solids are indeed more difficult to demonstrate for the reason that high-resolution NMR detection in solids requires fast spinning of the sample at the “magic” angle, a method used to suppress rank-2 anisotropic interaction tensors. Hence, light-irradiation, which is used to generate radical-pair intermediates, has to be performed in an arrangement, which also allows for magic-angle spinning (MAS). A further challenge is that spin-sorting mechanism that rely on differences in the chemical reactivity of the radical pair in its different electronic spin states to produce photo-CIDNP effects are not expected to be operative in solids. This is because the radical centers, which constitute the radical pair, cannot separate by molecular diffusion, as would be the case in liquids or solutions. For this reason, eventually all radical pairs would recombine, regardless of the nuclear spin state and CIDNP effects would disappear, at least in the steady-state.

Nonetheless, despite these issues, solid-state CIDNP effects have been observed experimentally and explained theoretically. For a long time, the theoretical explanation [6–9] has been based on several models, valid for different particular cases, which make use of special assumptions about either the chemical reactivity and fate of polarization in different reaction channels or the presence of anisotropic spin interactions. Until recently, such a treatment has remained disconnected from the well-established liquid-state CIDNP theory [10–15]. Our recent papers propose that CIDNP can be regarded from a general perspective, allowing one to use essentially the same approach for both liquids and solids. However, the solid-state case still remains more complex due to the presence of additional interaction terms, which average to zero in liquids. In this paper, we introduce the history of the solid-state photo-CIDNP effect, describe applications of photo-CIDNP MAS NMR, discuss the concepts of level crossings (LCs) and level anti-crossings (LACs) as well as provide an outlook on possible developments and open questions in the field.

## CIDEP, CIDNP and SCRPs in Liquid State

It might be due to the higher polarization of electrons compared to nuclei that advances in EPR have often preceded analogous developments in NMR. EPR was first demonstrated in 1944 by Zavoisky, a year before the first successful NMR were presented independently by Purcell and Bloch. Similarly, the phenomenon of chemically induced dynamic electron polarization (CIDEP) was reported before chemically induced dynamic nuclear polarization (CIDNP). In 1963, Fessenden and Schuler [16] reported unusual intensity patterns in the EPR signals of transient alkyl radicals produced by radiolysis. In particular, they observed emissive polarization from hydrogen and deuterium atoms in liquid methane solution at − 175 °C. The cause of the signal inversion, i.e., of the non-Boltzmann distribution over spin-levels, was unknown at that time, as they stated explicitly. In the same study, it was recognized that CIDEP of hydrogen cannot be observed under solid-state conditions as tested using frozen methane (mp − 183 °C) solutions.

In 1967, Bargon and Fischer reported CIDNP, a pattern of emissive and enhanced absorptive lines, upon heating of organic peroxides during a ^1^H liquid-state NMR experiment [17]. In the same year, these results were confirmed by Ward and Lawler [18]. A year later, Cocivera reported this phenomenon in a photochemical reaction and the term “photo-CIDNP” was coined [19]. The term CIDNP was based on the initial interpretation in terms of an Overhauser effect as “chemically induced DNP”, and the term CIDEP was created in analogy to CIDNP. The classical radical-pair mechanism (RPM) as proposed in 1969 by Kaptein and Closs independently [20, 21] allowed the thermal and light-induced CIDNP phenomenon to be explained on the basis of spin-sorting under liquid-state conditions: when the radical pair is in its singlet state, recombination is possible, while in the triplet state, recombination is impossible and the two radicals separate. In a coherently evolving radical pair, nuclear spin states control the electronic state and therefore the chemical fate. Hence, particular nuclear spin-states are enriched in the two products, distinguished by their chemical shifts, leading to a signal pattern which is explained by Kaptein’s sign rules [22], and their limits were discussed [23]. With the RPM, the field of spin-chemistry was born, stating that extremely weak nuclear Zeeman energies can kinetically control chemical reactions having significantly higher reaction energies: that is an impertinence for classical chemical-reaction theory. For the interpretation of CIDEP spectra, this theoretic concept has been adapted and the term “spin-correlated radical pair” (SCRP) was introduced [24, 25]. Some later break-throughs were studies in gas phase [26, 27], ^19^F photo-CIDNP NMR [28], usage of fluorescein as dye [29], studies to explore organic reaction mechanisms [30–32], photo-CIDNP-based studies of protein structures [33], time-resolved liquid-state photo-CIDNP studies on protein folding [34], the concept of isotropic mixing by Hans-Martin Vieth and co-workers [35–37] as well as field-cycling photo-CIDNP NMR of liquid samples [35, 38, 39]. Examples of molecular systems, which exhibit very strong liquid-state CIDNP effects, are given by confined SCRP, which is the case of radicals trapped in micelles [40, 41] and, in particular, biradical systems [42–44]. In the latter case, suitable systems can be either rigid [45, 46] or flexible [42–44, 47] biradicals.

## Spin-Dynamics in Photosynthetic Systems

CIDEP of SCRPs has been studied in particular on natural photosynthetic systems (for review, see [48, 49]). One might state that, at that time, the development of EPR methodology and the study of the light-induced steps of photosynthesis went hand in hand [50]: After a short light pulse, emissive EPR signals occur, which decay on the time-scale of the spin–lattice relaxation time *T*_1_. Such a flash-induced transient CIDEP signal was observed for the first time in 1975 from chloroplasts [51]. In addition, the triplet state of the donor, which can easily build up upon illumination at cryogenic temperatures, also shows a spin-polarized EPR spectrum [52].

Photosynthetic systems (for review, see [53]) also show strong magnetic field effects (for review, see [54]) on formation of both triplet and radical-pair states as observed in 1977 [55, 56]. It turned out that these effects are anisotropic, i.e., sensitive to molecular orientation [57]. Hence, the electron spin dynamics of photosynthetic reaction center proteins was well characterized using EPR methods (for review, see [49]). In particular, the advent of high-field EPR experiments resulted in significantly improved spectral dispersion (for review, see [58]). The study of the purple bacterial electron donor cofactors allowed its *g*-tensor [59] and the local electron spin density distribution [60] to be resolved. The latter study allowed the interpretation of photo-CIDNP ^13^C MAS NMR data [61] (see below) to be compared to results obtained with another spectroscopic method for the first time. Similarly, the plant RCs were explored by EPR methodology [62, 63]. The observation of quantum-beat oscillations by time-resolved EPR spectroscopy in 1994 [64] raised the question of the exact mode of electron-nuclear interaction in the early steps of photosynthetic electron transfer. There was, however, no successful NMR experiment, and the role of magnetic nuclei in the spin-machinery remained unclear.

## The First Successful Observation of the Solid-State Photo-CIDNP Effect

In 1983, optical nuclear polarization (ONP) work on molecular crystals [65] revealed the occurrence of radical pairs. Although in such systems, nuclear spin polarization is usually derived from the molecular triplet state, in some cases hydrogen-transfer in the triplet state can give rise to radical-pair formation and subsequent CIDNP of the ground state after recombination. However, such concepts have never been generalized and extended to other systems. A solid-state photo-CIDNP effect is also possible in so called “plastic crystals” [66–68], in which some rotational motions persist. However, this situation is very similar to the liquid-state case: due to molecular motion, CIDNP formed upon recombination of biradicals exhibits essentially the same features of polarization formation. In other cases, as explained in the introduction, “spin-sorting” arguments do not hold and the possibility of CIDNP formation in solids was not evident.

Around 1980, Hoff and Kaptein tried to observe photo-CIDNP by ^1^H liquid-state NMR at 360 MHz on illuminated photosynthetic reaction centers (Arnold Hoff, Robert Kaptein, personal communications to J.M.). These experiments were hampered by the large molecular weight of these proteins (around 100 kDa), which was far beyond the limit for fast isotropic motion needed for liquid-state NMR. In their 1982 review by Boxer et al. [69] is stated: “The only well-developed example of a radical-pair reaction in the solid state whose outcome can be influenced by magnetic fields comes from photosynthetic systems”. The authors were aiming to rationalize the anisotropic field-dependence of the triplet formation within the frame of the RPM by introducing orientation dependent interactions. In 1987, Goldstein and Boxer [6] proposed a modification of the RPM that could also occur in solid phase. If, after spin-sorting, the nuclear hyperpolarization of one branch is quenched and the nuclear hyperpolarization of the other branch lives longer (with normal nuclear *T*_1_), then transient nuclear hyperpolarization will be observable. This mechanism is called “cyclic reaction” in the liquid-state photo-CIDNP community [13–15, 70] and “differential relaxation” (DR) in the solid-state photo-CIDNP community. The target of Boxer and Goldstein’s theoretical analysis was the quinone blocked-bacterial photosynthetic reaction center in which light-induced cyclic electron transfer leads to formation of a SCRP that recombines to the donor triplet state. Spin relaxation in the triplet state destroys the nuclear hyperpolarization of the triplet decay pathway, while the nuclear hyperpolarization of the singlet state remains. The authors stated: “Our analysis suggests new classes of experiments and indicates the need to reinterpret some past experimental results”.

**Fig. 1:**
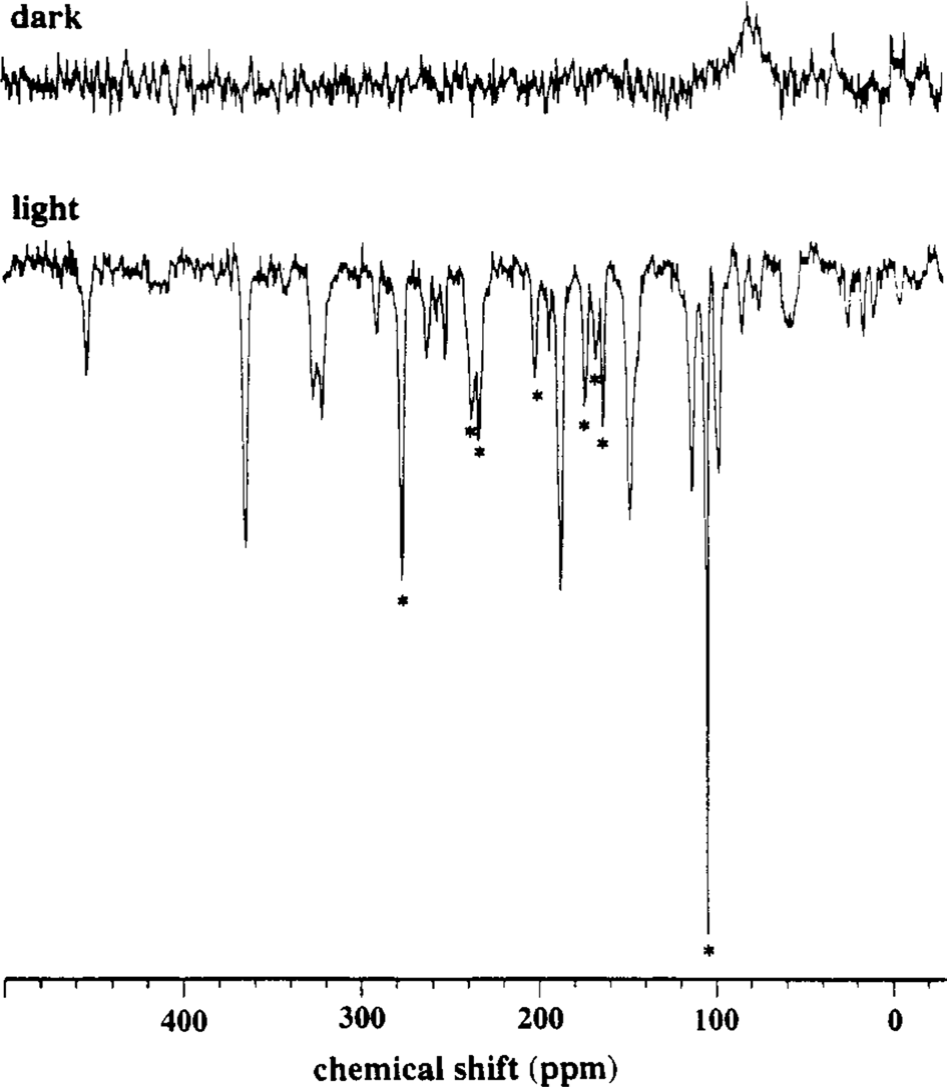
The first demonstration of the solid-state photo-CIDNP effect as published in 1994 by Zysmilich and McDermott. The ^15^N MAS NMR spectrum has been obtained from a ^15^N enriched, quinone-depleted and frozen sample of the isolated reaction center of the purple bacterium *Rhodobacter sphaeroides* under continuous illumination with white light at 9.4T. MAS frequency was 3600 Hz, and centerbands are marked with asterisks. Chemical shifts are given relative to an external reference of 1M ^15^NH_4_Cl in 2M HCl. This picture has been reproduced from [71] (Not subject to US copyright)

**Scheme 1:**
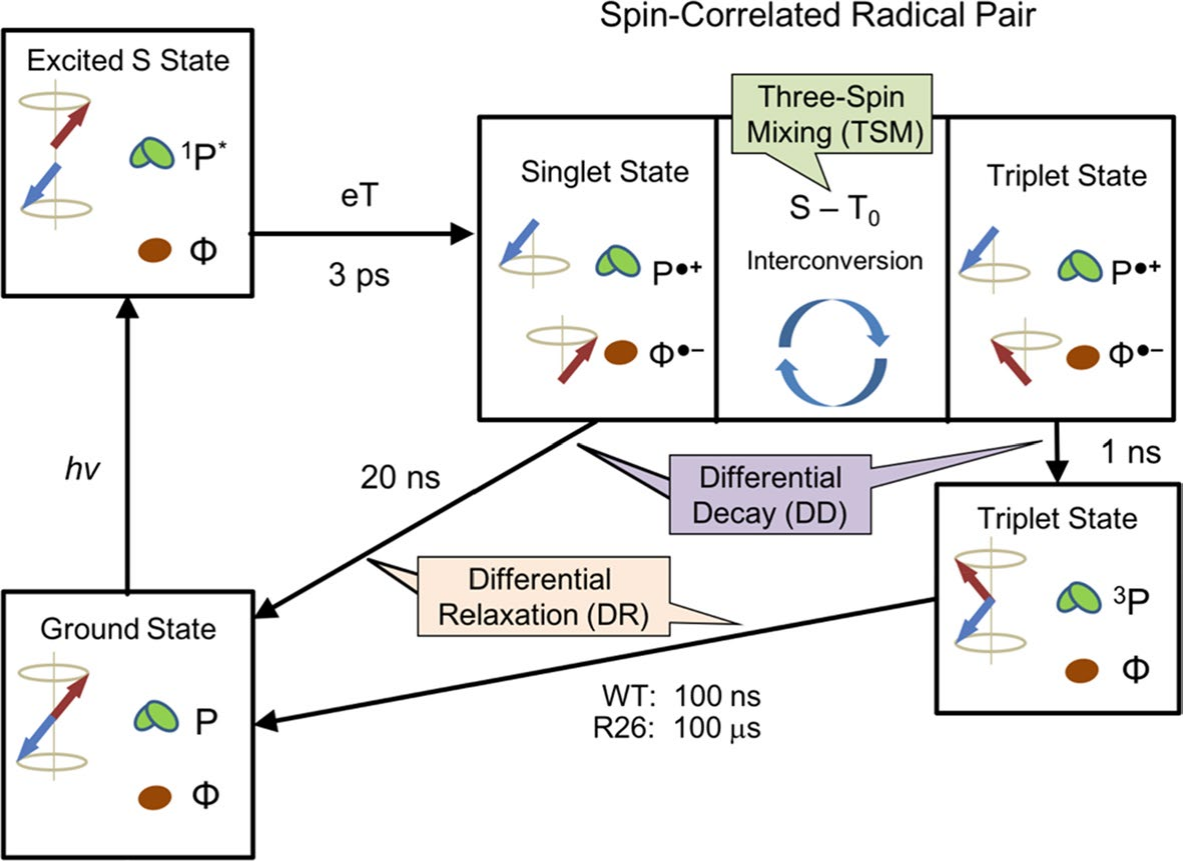
Photocycle occurring in quinone-deactivated photosynthetic reaction centers of the purple bacterium *Rhodobacter sphaeroides* wild type (WT) and the carotenoid-less mutant R26 [53, 79]. Upon light excitation, donor dimer, the so-called special pair, is excited from its electronic ground-state (P, green) to the first excited singlet state (^1^P*). Electron transfer (eT) occurs on picosecond time-scale and forms between P and primary acceptor BPhe (Φ, brown) a SCRP in its pure singlet state, ^1^[P^●+^---Φ_A_^●−^]. The singlet SCRP has two possible chemical fates: either it recombines to form the diamagnetic ground state or it undergoes ***S ↔ T***_***0***_ interconversion (at high fields) to convert into the triplet state of the SCRP, ^3^[P^●+^---Φ_A_^●−^]. The triplet-state SCRP is not allowed to directly recombine to form the electronic ground state. Instead, it undergoes back-eT to generate a triplet state on the special pair, ^3^P. In the WT, the lifetime of ^3^P is about ~ 100 ns, while in the R26 mutant the time scale is extended to ~ 100 μs due to the lack of a carotenoid. During the photocycle, the various mechanisms producing solid-state photo-CIDNP occur: Three-spin mixing (TSM), differential decay (DD) and differential relaxation (DR). The TSM occurs during singlet–triplet interconversion, the DD relies on the different concentration of both spin-states occurring during SCRP evolution, and in the DR the nuclear spin population related to the triplet channel is relaxed, while that of the singlet channel survives

It was due to the progress of biological solid-state NMR by adaptation of ^13^C and ^15^N magic-angle spinning NMR which allowed the nuclear spins in photosynthetic reaction centers to be probed. This fundamental breakthrough was achieved by Zysmilich and McDermott in 1994 [71] demonstrating that a photo-CIDNP effect is possible under solid-state conditions by illuminating quinone-blocked ^15^N-enriched bacterial photosynthetic reaction centers from the R26 mutant strain of *Rhodobacter sphaeroides* (Fig. 1). Subsequently the effect was also observed by ^13^C photo-CIDNP MAS NMR in bacterial reaction centers with ^13^C at natural abundance [72]. Originally, these first photo-CIDNP MAS NMR results were explained by the DR mechanism [73]. The fact that acceptor signals, which can be well distinguished in ^15^N NMR, also occurred sparked a new discussion of the exact origin of the polarization, and two new spin-chemical mechanisms were proposed (Scheme 1): (1) Based on the effect of the pseudosecular contribution to the hyperfine interaction on the free spin-evolution of the SCRP, Jeschke proposed a fully coherent transformation of nuclear coherences to nuclear polarization called three-spin mixing (TSM) [7]. Later, a low-field analogue of the TSM was proposed [74] and it has been demonstrated that this “solid-state mechanism” simply relies on a sufficiently high degree of orientation, which can also be provided by “liquid” membranes. Hence, a system can be in a “liquid-state regime” with respect to NMR and in a “solid-state regime” with respect to hyperfine anisotropies [75]. (2) Polenova and McDermott proposed a mechanism relying on the difference of the decay rates of the different spin-states of the SCRP, allowing for selective enrichment of particular nuclear spin-states, called differential decay (DD) [8]. When it was realized that all three mechanisms might be active in parallel, sign rules were formulated and it was stated that, in contrast to liquid-state photo-CIDNP, for the solid-state photo-CIDNP effect, the field-dependent maximum depends on the gyromagnetic ratio γ of the observed nucleus [9]. More recently and in-line with field-cycling MAS NMR data [76], Ivanov and coworkers reformulated, unified and extended the theory in terms of level-crossings and level-anti crossings (see below) [77, 78].

## Studies on Bacterial Photosynthetic Reaction Centers

Reaction centers of the very well characterized purple bacterium *Rhodobacter sphaeroides* (for review, see [53, 79]) have provided the “gold-standard” for the development of the method. One of the long-standing questions concerning this reaction center is its functional asymmetry, i.e. having electron transport with 100% selectivity into almost structurally identical branches of cofactors. In an attempt to provide an answer, analysis by photo-CIDNP MAS NMR revealed three aspects: (1) The chemical shifts of both bacteriochlorophylls forming the special pair donor are readily distinguished in the electronic ground state [80, 81], (2) This asymmetry, is presumably due to different tuning by the substituents of the macrocycles [82], (3) the size of the orbital factors, i.e., of the orbital lobes, in the excited state are larger towards the active branch [83], dynamics studies indicated a difference between both halves [84]. Further studies were dedicated to several other bacteria, e.g., green-sulfur bacteria [85] and heliobacteria [86].

## Studies on Photosynthetic Reaction Centers of Plants, Algae and Diatoms

**Fig. 2:**
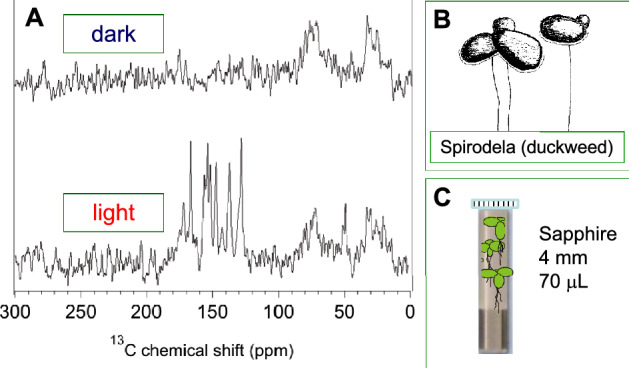
**a** The ^13^C solid-state photo-CIDNP effect has been observed by MAS NMR in entire plants of the aquatic plant *Spirodela oligorrhiza* (duckweed) obtained under continuous illumination with white light at 4.7 T [92]. While in the dark experiment solely signals from aliphatic carbons are detected (top), upon illumination light-induced signals appear in the aromatic spectral range (bottom). For the experiment, **b** full plants were inserted into **c** an optically transparent MAS rotor. The tetrapyrrole cofactors were selectively ^13^C-isotope enriched by feeding with ^13^C_1_-4-δ-aminolevulinic acid and the sample was pre-reduced with Na_2_S_2_O_3_

Photo-CIDNP MAS NMR on plants was reviewed recently [87]. Very briefly, both reaction centers, called photosystems I and II, were addressed with photo-CIDNP MAS NMR in various plants [88, 89]. Here, the question of the extremely high redox potential of photosystem II was addressed suggesting a chlorophyll-histidine complex acting as donor [90]. In photosystem I, the activities of the two branches of cofactors were explored [91]. Photo-CIDNP MAS NMR allowed signals from ^13^C labels introduced into reaction centers to be detected directly from entire plants (Fig. 2). The chemical shifts of these nuclei are very similar as those of isolated reaction centers [92]. Recently, reaction centers of diatoms were also studied and found to be similar to those of plant systems, especially in their electronic ground-state structure [93]. Remarkably, all natural photosynthetic systems tested showed the solid-state photo-CIDNP effect, while so far artificial photosynthetic systems have failed to display similar effects. Therefore, it has been speculated whether the occurrence of the effect correlates with efficiency [94].

## Studies on Flavoproteins

**Fig. 3:**
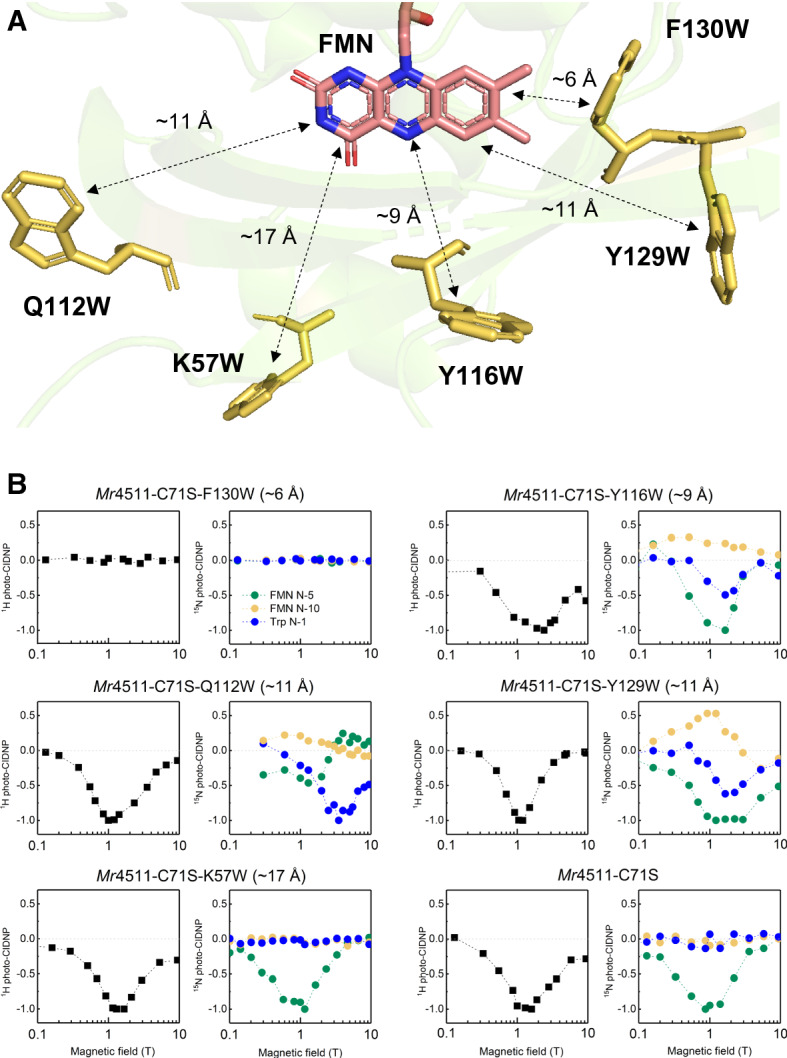
**a** Structure model of the flavoprotein named 4511 from *Methylobacterium radiotolerans* (*Mr*4511), a LOV domain which does not contain a tryptophan residue in its wild-type. For the photo-CIDNP NMR experiments, the functional cysteine residue in the domain was mutated into serine (*Mr*4511-C71S). In the *Mr*4511-C71S, one tryptophan was introduced to different positions, producing five additional mutants which allow for formation of light-induced SCRPs having different distances between the flavin and the introduced tryptophan ranging from ~ 6 and ~ 17 Å. All samples were uniformly ^15^N enriched. **b** The magnetic-field dependencies of photo-CIDNP formation detected by ^1^H and ^15^N NMR. While at short distance (~ 6 Å) no photo-CIDNP is detected, it occurs in the range of ~ 9 to ~ 11 Å. The differences observed between the two mutants having a distance of ~ 11 Å suggest, in addition to a distance dependence, an orientation dependence of the donor and acceptor units. Interestingly, at longer distance (~ 17 Å) and in the cysteine-less mutant without tryptophan, same patterns occur implying that other amino acids, assumably tyrosines, take over the role of the electron donor [98]

In 2005, Weber and coworkers observed ^13^C photo-CIDNP by liquid-state NMR on a so-called LOV-domain flavoprotein with the molar mass of around 15 kDa [95]. The authors identified that the SCRP is formed by the flavin and a tryptophan residue. Upon illumination, the flavin forms a triplet and attracts an electron from the aromatic amino acid, i.e., here the acceptor is photoexcited. A very similar LOV-domain showed strong photo-CIDNP in the frozen state measured by ^13^C MAS NMR [96]. Recently, a LOV domain was studied by field-cycling ^1^H, ^13^C and ^15^N liquid-state photo-CIDNP NMR, and the theoretical analysis revealed that the production of hyperpolarization is based on a solid-state mechanism. The most salient feature of this CIDNP formation in solids, which is different from that in liquids, is that the efficiency of formation and magnetic field dependence of the CIDNP are sensitive to the nuclear gyromagnetic ratio (see above). For this reason, the field dependences of different nuclei, e.g., of ^1^H, ^13^C and ^15^N, exhibit maxima at different magnetic fields providing evidence that the origin of the nuclear hyperpolarization is a solid-state photo-CIDNP mechanism [97]. These results also show a ^1^H solid-state photo-CIDNP effect for the first time although it is observed by liquid-state NMR because proteins tumble slowly and anisotropic interactions are not fully averaged on the timescale of the SCRP lifetime. Hence, the boundary between liquid and solid states blurs. Furthermore, flavoproteins allow properties of the SCRP such as the distance between the radicals and their orientations (Fig. 3) to be customized, opening again “new categories of experiments” [98].

## Theory Relying on Level Crossings and Level Anti-crossings

As mentioned above, formation of CIDNP in solids is more complex than in liquids, because (1) the radical centers cannot diffuse apart and (2) non-averaged anisotropic interactions are present, which are zero in the liquid state. Consequently, in order to account for experimental observations, it has become necessary to propose mechanisms like DR, TSM and DD. There have been, however, some experimental observations, that have challenged the completeness of the trio of mechanisms and increased the need to reduce the dependence on numerical calculations of polarization, since such an approach is complex due to the large number of parameters (reaction rates and magnetic interactions in the SCRP). First, a detailed field-cycling MAS NMR study of the solid-state photo-CIDNP effect showed an unexpected sign change of the nuclear polarization at low fields [76]. A sign change of the solid-state photo-CIDNP effect in the diatom *Phaeodactylum tricornutum* was also observed at higher magnetic fields and has not yet been rationalized [93]. Second, sign changes related to selective ^13^C isotope incorporation shifting the ratio between enhanced absorptive and emissive intensities in favor of the former have been observed in reaction centers of heliobacteria [86, 99] and photosystem I [91]. Hence, it appears that magnetic ^13^C isotopes are not “innocent” but involved into the spin-chemical machinery producing the hyperpolarization. Effects of magnetic isotopes on chemical reaction dynamics have been reported by Buchachenko, Sagdeev and Turro [100–103]. These works report magnetic isotope effects (MIEs) in liquid-state reactions, whereas MIE in the solid state, where anisotropic hyperfine interactions come into play, still needs to be investigated. When MIEs in solids are understood, the next step is to investigate the mutual effects of ^13^C nuclei on CIDNP formation. In liquids, such effects are well-documented: the presence of multiple nuclei strongly modifies the CIDNP field dependence and can even give rise to a change of the sign of the polarization (violation of the CIDNP sign rules) [23]. Finally, there is a need to explain the solid-state photo-CIDNP effect in flavoproteins in which the SCRP is triplet born and DR and DD are difficult to implement.

**Fig. 4:**
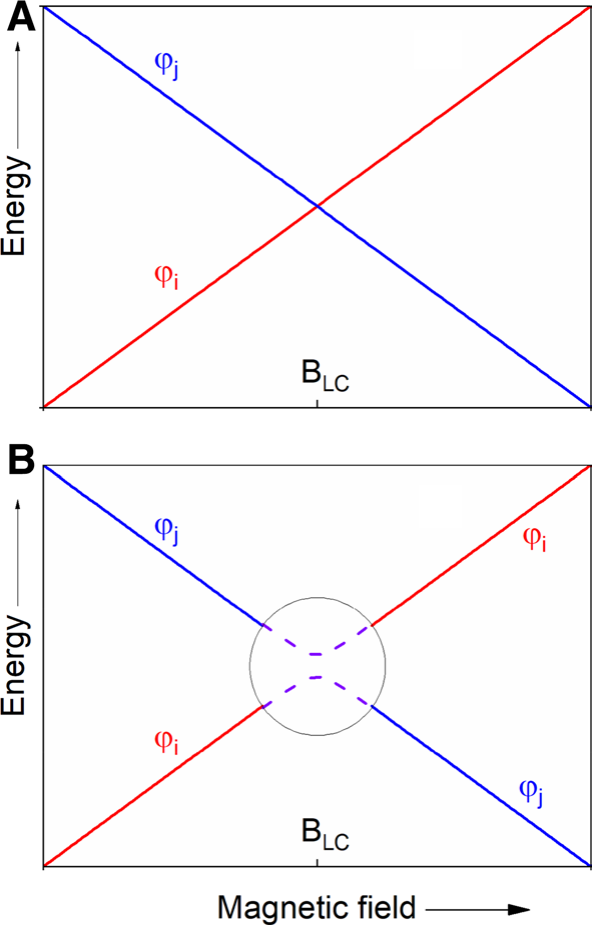
Energy level diagrams showing the magnetic-field dependence of the two spin states |φ_i_⟩ and |φ_j_⟩. **a** If the coupling matrix element V_ij_ = 0, i.e., there is no perturbation between both states, therefore both states undergo Level Crossing (LC) at the magnetic field B_LC_. **b** When the V_ij_ ≠ 0, the perturbation term V_ij_ can mix the states |φ_i_⟩ and |φ_j_⟩, and the LC is transformed into a Level Anti-Crossing (LAC). The minimal splitting of the LAC is 2V_12_

The language of LCs and LACs (Fig. 4) was introduced in 1929 by von Neumann and Wigner to connect quantum theory and thermodynamics  [104] and has also become popular in photochemistry [105]. Its application to CIDNP theory has the advantage that the concept has already been successfully applied for the interpretation of other hyperpolarization methods such as para-hydrogen polarization (PHIP) and optical nuclear polarization (ONP) (for review, see [106, 107]) and might evolve into a common hyperpolarization theory.

LCs between quantum states $|i\rangle$ and $|j\rangle$ become relevant in a special case when the initial SCRP state is a coherent state with the coherence between the states $|i\rangle$ and $|j\rangle$ being non-zero. The most common example is given by the situation where the SCRP is created at high magnetic field in the singlet state, whereas the eigenstates of its spin Hamiltonian are the Zeeman states $|\alpha \beta \rangle$ and $|\beta \alpha \rangle$ (as usual, $|\alpha \rangle$ and $|\beta \rangle$ denote the states with the quantum number m_s_ equal to + 1/2 and − 1/2, respectively). Under such conditions, the populations of the Zeeman states are equal, but coherence between them is present. This coherence evolves with time in an oscillatory way, giving rise to reversible transitions between the singlet state and the central triplet state. The frequency of the oscillations (hence, the rate of singlet–triplet transitions) is proportional to the splitting between the $|\alpha \beta \rangle$ and $|\beta \alpha \rangle$ states. This splitting is minimal (it is equal to zero) at the LCs, where the singlet–triplet transitions can no longer occur. LCs occur in specific nuclear spin states when the electronic $\Delta g$-term is matched to the hyperfine term: under such conditions, the singlet–triplet transitions in the corresponding state is turned off. As a consequence, nuclear spin sorting becomes most efficient and a maximum in the CIDNP field dependence can be observed.

If there is a perturbation term ${V}_{ij}$, which can mix the states $|i\rangle$ and $|j\rangle$, the crossing is “avoided”, being turned into a LAC. Under such conditions, coherent mixing of the states $|i\rangle$ and $|j\rangle$ occurs, giving rise to the transfer of population between them. Such a situation is relevant for liquid-state CIDNP formed at low magnetic fields. For instance, the S–T_±_ CIDNP mechanism is due to spin mixing at a LAC [1, 108, 109].

In solids, the same concept can be used to interpret the experimental observations. In particular in solids, LCs are very rare, because of the presence of various anisotropic interactions, which can mix almost any two states. Hence, some of the LCs, which have no effect in the isotropic case, are turned into a LAC and become important. An example is given by the crossing between $|\alpha \beta {\alpha }_{N}\rangle$ and $|\alpha \beta {\beta }_{N}\rangle$, which can be turned into a LAC by anisotropic pseudo-secular hyperfine terms, giving rise to a feature in the CIDNP field dependence (the subscript “$N$” denotes the nuclear spin state). The matching condition for the LC (consistent with the DD mechanism) corresponds to matching of the isotropic hyperfine term and nuclear Zeeman interaction, i.e., it is different for the nuclei with different gyromagnetic ratios. By analyzing LACs in the SCRP, it is thus possible [77] to determine the positions of the features in the CIDNP field dependence, and to obtain the polarization sign rules, in the spirit of classical Kaptein’s rules [22], well-known in the case of liquid-state CIDNP. The same method can be used [78] to analyze the most complex TSM case, and to obtain the matching conditions as well as the sign rules. Furthermore, other situations, like the inverted $\Delta g$-case, can be treated with this approach. Level-crossing analysis was applied for explanation of the CIDNP field dependence for photosynthetic reactions centers [78] (a case where the CIDNP sign change has been reported) and flavoproteins [97]. In the latter case, level crossing analysis confirmed that the magnetic fields corresponding to maximal polarization are different for different nuclei, such as ^1^H, ^13^C and ^15^N, implying a solid-state photo-CIDNP mechanism.

## Photo-CIDNP MAS NMR as an Analytical Method

Along with the elaboration of the theory, the solid-state photo-CIDNP effect has been developed into an analytical method allowing the cofactors carrying the SCRP and their environment to be explored (for review, see [110]). Presently, the following parameters can be obtained: (1) The chemical shifts observed in photo-CIDNP MAS NMR experiments refer to the electronic structure after the photo-cycle, i.e., after recombination. So far as chemical shifts of the compounds in the dark are known, no light-induced shifts are observed [80]. Chemical-shift assignments can be obtained by RFDR, DARR or INADEQUATE experiments [111, 112] in which the initial cross-polarization step is exchanged by a pulse on the ^13^C channel. (2) The solid-state photo-CIDNP intensities obtained under continuous illumination are composed of up to three mechanisms (TSM, DD and DR) and their contributions might be difficult to disentangle. In bacterial reaction centers of wild type *Rhodobacter sphaeroides*, the DR does not occur, due to the presence of carotenoids. Theoretical analysis explains the entirely emissive spectral envelope by the dominance of the TSM over the DD. In the carotenoid-free mutant R26, enhanced absorptive signals occur selectively from the donor. The TSM intensities can be correlated to local electron spin densities in the p_z_ orbitals [113] within the SCRP, while DR intensities are related to the local electron spin densities in the donor triplet state allowing the electronically excited state to be reconstructed [83]. (3) The initial solid-state photo-CIDNP intensities obtained under nanosecond laser-flash illumination arise from spin sorting, which is related to the isotropic hyperfine interaction and provides access to the total local electron spin density [114, 115]. (4) Nanosecond laser-flash kinetic experiments provide information about the lifetime of the radical pair, the donor triplet state as well as the *T*_1_ values of individual nuclei. In ^13^C isotope labelled samples, the polarization build-up dynamics by spin-diffusion can be followed as well [116]. (5) Lineshape and linewidth, as in normal NMR, refer to local dynamics and order. (6) Photo-CIDNP DIPSHIFT MAS NMR provides the local dipolar field for each labelled heavy-atom position, which can act as a local reporter for mobility [117]. (7) Photo-CIDNP spin-torch MAS NMR experiments transfer the hyperpolarization from ^13^C and ^15^N nuclei into the surroundings allowing tuning effects by the protein matrix to be studied [118].

## Outlook

The discovery of the solid-state photo-CIDNP effect in flavoproteins was of great relevance since these light-driven spin-machines can be manipulated more easily and are more versatile than photosynthetic systems. In particular, the position of the electron donor can be changed to almost any position of the protein by mutagenesis. Furthermore, these proteins can be expressed in *E. coli* and (at least the amino-acid positions) can be isotope labelled straightforwardly. Such modifications might allow the solid-state photo-CIDNP effect to be optimized for specific magnetic field strengths. It will be possible to mimic such systems in artificial flavoproteins. Proteins to which a surface-selective anchor is attached, might allow biomedical imaging experiments analogous to those using fluorescent labels such as green fluorescent protein in optical microscopy.

## Abbreviations

CIDEP: Chemically induced dynamic electron polarization
CIDNP: Chemically induced dynamic nuclear polarization
DARR: Dipolar assisted rotational resonance
DD: Differential decay
DIPSHIFT: Dipolar chemical shift correlation
DNP: Dynamic nuclear polarization
DR: Differential relaxation
EPR: Electron paramagnetic resonance
eT: Electron transfer
INADEQUATE: Incredible natural abundance double quantum transfer experiment
LC: Level crossing
LAC: Level anti-crossing
LOV: Light-oxygen-voltage (sensing domain)
MAS: Magic angle spinning
MIE: Magnetic isotope effect
mp: Melting point
NMR: Nuclear magnetic resonance
ONP: Optical nuclear polarization
RFDR: Radio-frequency driven recoupling
RPM: Radical pair mechanism
SCRP: Spin-correlated radical pair
TSM: Three-spin mixing
